# Exercise fatigue diagnosis method based on short-time Fourier transform and convolutional neural network

**DOI:** 10.3389/fphys.2022.965974

**Published:** 2022-08-30

**Authors:** Haiyan Zhu, Yuelong Ji, Baiyang Wang, Yuyun Kang

**Affiliations:** ^1^ School of Physical Education and Health, Linyi University, Linyi, China; ^2^ School of Information Science and Engineering, Linyi University, Linyi, China; ^3^ School of Life Science, Linyi University, Linyi, China

**Keywords:** exercise fatigue, ECG, STFT, CNN, health

## Abstract

Reasonable exercise is beneficial to human health. However, it is difficult for ordinary athletes to judge whether they are already in a state of fatigue that is not suitable for exercise. In this case, it is easy to cause physical damage or even life-threatening. Therefore, to health sports, protecting the human body in sports not be injured by unreasonable sports, this study proposes an exercise fatigue diagnosis method based on short-time Fourier transform (STFT) and convolutional neural network (CNN). The method analyzes and diagnoses the real-time electrocardiogram, and obtains whether the current exerciser has exercise fatigue according to the electrocardiogram. The algorithm first performs short-time Fourier transform on the electrocardiogram (ECG) signal to obtain the time spectrum of the signal, which is divided into training set and validation set. The training set is then fed into the convolutional neural network for learning, and the network parameters are adjusted. Finally, the trained convolutional neural network model is applied to the test set, and the recognition result of fatigue level is output. The validity and feasibility of the method are verified by the ECG experiment of exercise fatigue degree. The experimental recognition accuracy rate can reach 97.70%, which proves that the constructed sports fatigue diagnosis model has high diagnostic accuracy and is feasible for practical application.

## 1 Introduction

In recent years, competitive sports have increasingly developed, and many people have gradually realized the importance of sports to health and began to exercise regularly in their daily lives. However, it is not uncommon for ordinary people and professional athletes to be injured during sports, and most of them are caused by physical fatigue due to inappropriate exercise methods and inappropriate exercise intensity (Campbell et al., 2017; Evans et al., 2022). Reasonable physical activity is good for health, but unreasonable high-intensity exercise will lead to excessive fatigue, which will cause physical damage, and even cause tachycardia and myocardial strain in severe cases, which will eventually endanger life. How to judge the degree of fatigue of a person’s exercise and make a reminder when the degree of fatigue is serious has become a very important issue (Pernek et al., 2015; Tang et al., 2020; Liu et al., 2021; MacIntosh et al., 2021). In most cases, exercisers judge the fatigue level of the current activity by observing their breathing rate, heart rate and other system indicators and exercise duration, which is highly subjective. Respiration rate, heart rate and other signals are only rough information, and the accuracy of monitoring the human state during exercise is not high. Exercise duration on the other hand only applies to sustained aerobic activities like track and field or cycling. For discontinuous and fixed-position training like weight-lifting, indicators such as respiratory rate, heart rate and exercise duration are not applicable, and it is difficult to obtain an accurate result only with them (Gatti et al., 2014; Yang et al., 2019; Huang et al., 2020; Gundogdu et al., 2021).

In recent years, researchers have turned their attention to other more informative physical indicators for detecting fatigue during exercise (Garcia-Garcia et al., 2016; Chai et al., 2019; Yang et al., 2019). Padhmashree and Bhattacharyya, (2022) used multi-channel EEG signals and extract features for emotion recognition. Lakhan et al. (Sharma and Bhattacharyya, 2021; Sharma et al., 2021; Sharma et al., 2022) used EEG and wavelet transform to identify the stress of the human body and produced good results. Sugay et al. (Galen and Malek, 2014) proposed an electromyogram to monitor neuromuscular fatigue during sustained exercise. Kulawiec et al. (2021) used instruments to evaluate the blood sugar level of the human body after exercise to judge the fatigue degree of the body. Pero et al. (2020) monitor the body’s urine composition after exercise, and monitor the fatigue status of athletes according to the composition of urine before and after exercise. Xiao-qiu et al. (Wang and Yin, 2019) used sensors to collect physiological signals such as heart rate, ventilation times and oxygen uptake, and then used machine learning methods to analyze them, achieving better detection results. Cui Juan et al. (Wang, 2022) used comprehensive health monitoring technology to diagnose exercise fatigue in aerobics, and obtained an accuracy of 85% in the fatigue diagnosis of this exercise. You-Lei et al. (Fu et al., 2022) used deep learning technology to identify the features of surface electromyography, which was used for the fatigue level of sitting office. Abid et al. (Minhas et al., 2022) collected the driver’s facial image and assessed the driver’s fatigue level to prevent the occurrence of traffic accidents. Muhammad et al. (Usman et al., 2022) used an IoT system to collect EMG and observe muscle contraction and fatigue analysis. It can be seen that the more signal information, the better the monitoring effect will be. However, most of these traditional methods are only targeted at a specific movement. When the algorithm runs in a more complex and diverse environment, traditional machine learning algorithms such as SUPPORT vector machine (SVM), K-nearest Neighbors (KNN) are difficult to adapt to all environments with one algorithm. With the advent of wearable devices, monitoring devices have become miniaturized and can obtain ECG signals during exercise in real time. The researchers preprocessed the ECG signals using different transformations, and then combined with the shallow machine learning model, and achieved certain results, and the real-time monitoring was guaranteed, but there are still problems such as incomplete feature extraction, low accuracy, and poor generalization ability. With the development of artificial intelligence, researchers have proposed different real-time monitoring systems based on deep learning technology to identify sensor signals to obtain the fatigue state of the human body in motion. Jian et al. (Yu, 2021) used a convolutional neural network-based motion recognition technique to identify training fatigue levels. Vahid et al. (Farrahi et al., 2020) used machine learning algorithm to identify the joint acceleration collected by multiple sensors and judge the motion fatigue degree, with an accuracy of 80.4–90.7% on different data sets. The gradual maturity of technology based on Internet of Things (IoT) and wearable technology. AFZAAL et al. (Hussain et al., 2021) recorded signals such as electrocardiogram, heart rate, and respiratory rate during exercise. According to these signals, the system could identify the physical state of athletes with 97% accuracy.

Most of these studies have used electrocardiographic signals (ECGs) from the heart, which contain rich exercise-related features, and high diagnostic accuracy has been achieved based on these features. The miniaturized ECG monitor has a small size and can be carried around during daily exercise training. Exercise is a continuous process, and it is not comprehensive to analyze the state of exercise fatigue only based on a certain moment. There is a close relationship between the fatigue state of the human body at different times (Schiphof-Godart et al., 2018; Guan et al., 2021; Shi, 2021). CNN is a feedforward neural network, which includes deep structure and convolution computation.

Therefore, this paper proposes an exercise fatigue diagnosis method based on short-time Fourier transform and convolutional neural network, and monitors the fatigue degree of the human body during exercise according to the ECG signal. Firstly, the Visual Geometry Group (VGG) convolutional neural network model used for training was established, and the attention mechanism imitating biological eye attention behavior was added into the model to improve accuracy; then the signals collected by the sensor are converted into spectrograms using STFT, and the set divided into training sets and validation sets. The set is fed to the VGG network for training and recognition, and an accuracy of 99.9% is obtained. The experimental results show that the real-time ECG signal can be used to monitor the fatigue degree during exercise with high accuracy. On the other hand, the identification data only needs to be derived from the spectrogram by STFT, and no additional data preprocessing is required. The spectrogram is directly sent to the neural network for classification and recognition, which has better robustness and generalization ability.

## 2 Methods

In this part, short-time Fourier transform and convolutional neural network will be briefly introduced to diagnose fatigue degree in motion, mainly including the structure of VGG neural network and the principle of ECG signal processing as spectrum graph. In other parts of this section, the evaluation index of neural network model and the overall method flow of using ECG signal to diagnose exercise fatigue degree are also introduced.

### 2.1 Short time Fourier transform

STFT is a commonly used signal processing method to quantify the time-varying frequency and phase content of a nonstationary signal. By adding a window function (the length of the window function is fixed), the time domain signal is firstly windowed, and the original time domain signal is divided into multiple segments by sliding windows, and then each segment is STFT transformed to obtain the signal time spectrum. Time and frequency are expressed by STFT as two-dimensional functions, as follows: f(t) is the time domain signal, and g(t−τ) is the time window centered at time τ . It can be seen from this that STFT is the Fourier transform of the signal f( t ) multiplied by a window function g( t−τ) centered on τ (Munteanu et al., 2009; Wang et al., 2020). The calculation formula of STFT is shown in Formula 1 as shown below.

$$STFT_{f}{\left(t,\omega \right)}_{\;}=\overset{+\infty }{\underset{-\infty }{\int }}f\left(t\right)g\left(t-\tau \right)e^{-j\omega t}dt$$ (1)

### 2.2 Convolutional neural network

The most basic CNN model consists of four parts, including convolution layer, pooling layer, full connection layer and classification layer. The convolution layer uses convolution kernels to extract features and contains multiple convolution kernels. Each neuron in the network maintains a one-way connection with the neuron at the next layer, which is called “receptive field”, and the size of this area depends on the size of the convolution kernel. Formula 2 is defined as follows: the gray value of the image is represented by v ; the size of the convolution kernel is the value of p × q ; the weight of the convolution kernel is represented by w ; the bias b is added after the convolution; f is the activation function.

$$z_{x,y}=f\left(\overset{p\text{∗}q}{\underset{i}{\sum }}w_{i}v_{i}+b\right)$$ (2)

The pooling layer is a subsampling operation, and its main objective is to gradually reduce the number of features contained in the feature graph. In this paper, Max Pooling is used, and its function is Formula 3.

$$f=Max\left(x_{m,n},x_{m+1,n},x_{m,n+1},x_{m+1,n+1}\right)\;\left(0\leq m\leq M,0\leq n\leq N\right)$$ (3)

The fully connected layer reintegrates the local features extracted in the previous steps. In the following Formula 4, $\theta_{1},\theta_{2},\ldots ,\theta_{k}$ is the learning parameter of the model, and multiplication by $\frac{1}{\sum_{j=1}^{k}e^{\theta_{j}^{T}x_{i}}}$ is to make the probability distribution between [0, 1].

$$h_{\theta }\left(x_{i}\right)=\left[\begin{array}{c} p\left(y_{i}=1\text{|}x_{i};\theta \right)\\ p\left(y_{i}=2\text{|}x_{i};\theta \right)\\ \vdots \\ p\left(y_{i}=k|x_{i};\theta \right) \end{array}\right]=\frac{1}{\sum_{j=1}^{k}e^{\theta_{j}^{T}x_{i}}}\left[\begin{array}{c} e^{\theta_{1}^{T}x_{i}}\\ e^{\theta_{2}^{T}x_{i}}\\ \vdots \\ e^{\theta_{k}^{T}x_{i}} \end{array}\right]$$ (4)

The classification layer (SoftMax) is a general form of logistic regression, which can implement multi-classification problems. The input data has k categories, and the probability of input data x belonging to each of k categories is calculated using the SoftMax function.

**FIGURE 1:**
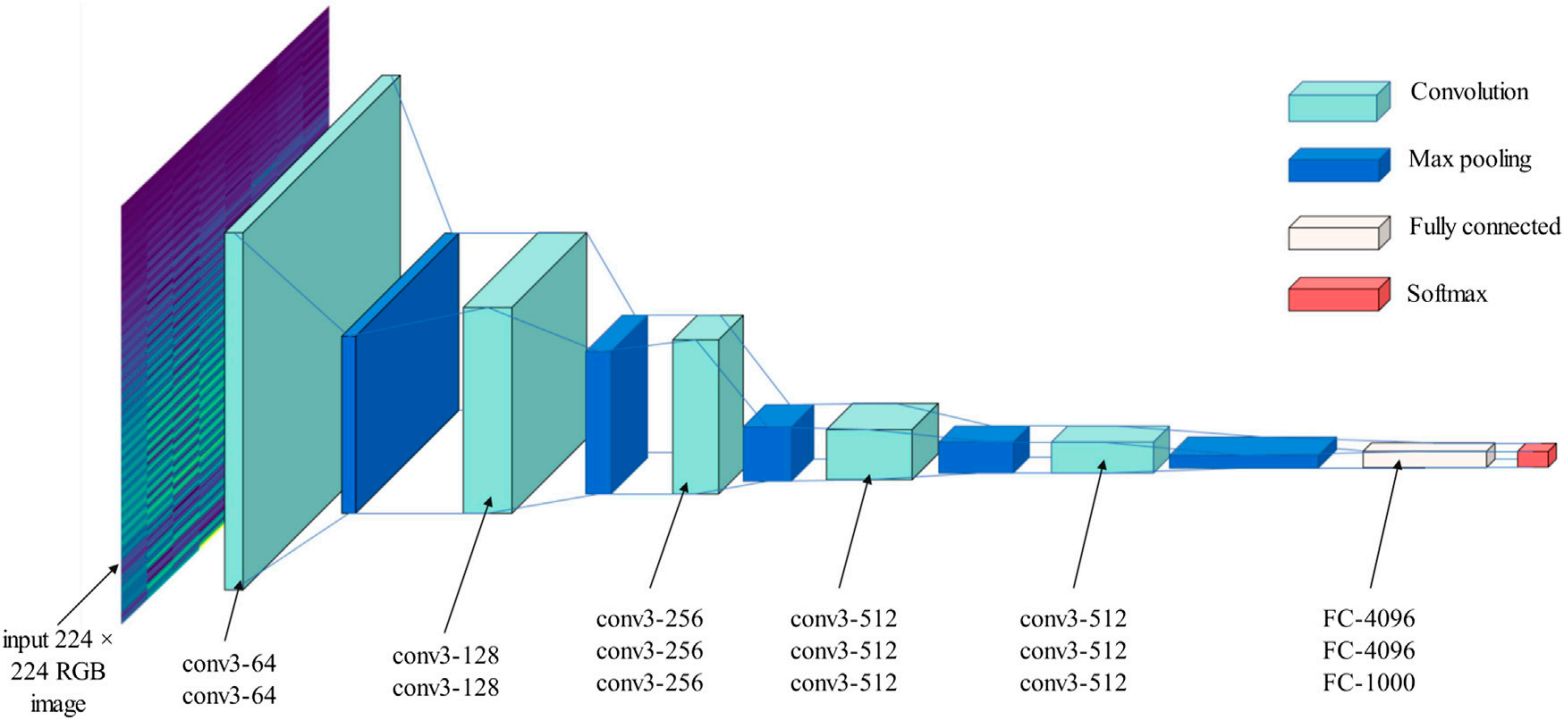
Structure of VGG convolutional neural network.

The mainstream CNN models include AlexNet, GoogLeNet and VGG network. In 2014, the Visual Geometry Group at the University of Oxford proposed an even deeper neural network with 13 convolutional layers and three fully connected layers. The proposed network structure is stacked by multiple 3×3 convolutional layers. Multiple small convolutional kernels can replace the traditional large convolutional kernels to complete the required calculation and reduce the amount of calculation. In practice, the superposition of two small 3×3 convolutional kernels can replace a 5×5 large convolutional kernels, and the superposition of three small convolutional kernels can replace a 7×7 large convolutional kernels (Simonyan and Zisserman, 2014). The network structure of VGG is shown in Figure 1.

**FIGURE 2:**
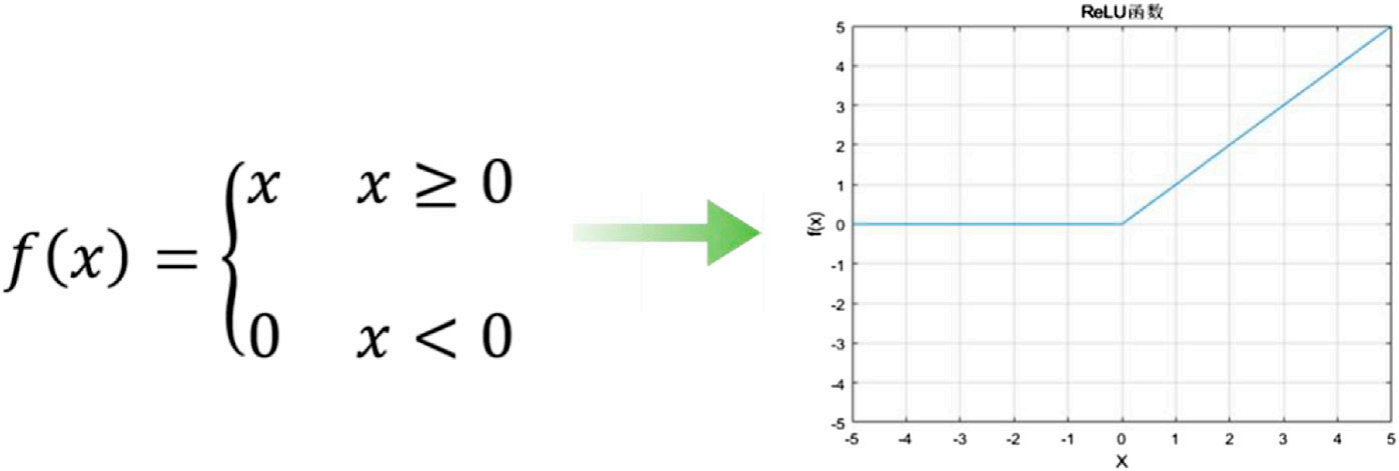
ReLU function and function image.

The pooling window of the pooling layer has a size of 2×2 and a step of 2. In the fully connected layer, it is combined by 3 consecutive full connections. The first two channels are 4096 and 4096 respectively, and finally the classification output is performed by the SoftMax classifier with 1000 labels. The network structure is shown in the figure above. VGG-16 uses the ReLU activation function. Using the sparse running results of ReLU function, the backpropagation errors can be effectively reduced in the training process, and the convergence speed of neural network can be accelerated Figure 2.

The VGG network is used to build a signal recognition model. The activation function of the model uses the ReLU function. The number of weight parameters of VGG-16 is as high as 70303557, of which the three fully connected layer parameters account for a large proportion. The original parameter setting of VGG-16 is to complete 1000 classification, and the classification of processing signals is less. Therefore, the first two fully connected layers only use half of the original number of nodes, that is, 2048 nodes, and the third fully connected layer has 6 nodes corresponding to the classification category, which further improves the training efficiency and recognition accuracy of the neural network model.

### 2.3 Evaluation indicators

#### 2.3.1 Loss function and accuracy function

In the process of continuous training, CNN needs an evaluation index to evaluate the training effect of the current CNN model and decide whether to terminate the training. In general, loss function and accuracy function are used to evaluate the model. The smaller the loss function is, the higher the accuracy value is, and the better the model effect is. In network constructed in this paper using the evaluating model of cross entropy loss function, this kind of loss function is suitable for the multiple classification problems. Formula 5 as shown below, which *m* represents the number of categories, y is a symbolic function and value of 0 or 1. When the model prediction probability true value is 1, false prediction probability value is 0. The prediction probability of sample i  predicted by the model as class c is represented by $p_{ic}$ .

$$Loss=\frac{1}{n}\underset{i}{\sum }\overset{m}{\underset{c=1}{\sum }}y_{ic}\log\left(p_{ic}\right)$$ (5)

Accuracy is usually used to evaluate the evaluation results of the current model in the test set during model testing. In multiple categories, a model that identifies a category correctly will be defined as positive, and a model that identifies a category as any other category will be defined as negative. The formula is defined as Formula 6, FP represents the number of other categories that are predicted to be the current category; TP represents the number of categories that are predicted to be correct; FN represents the number of current categories that are predicted to be other categories; FP represents the probability that other categories are predicted to be correct.

$$Accuracy=\frac{TP+TN}{TP+TN+FP+FN}$$ (6)

#### 2.3.2 Confusion matrix

**FIGURE 3:**
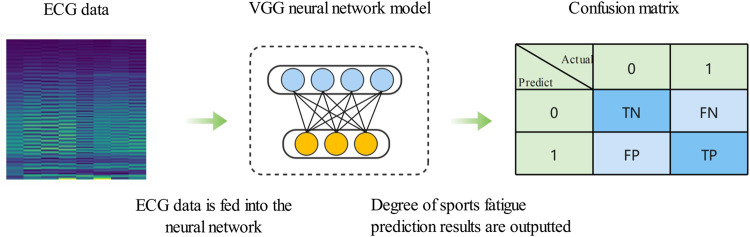
Confusion matrix visualization.

Visualization method is simple while intuitive observation model prediction results, the multiple classification problems, and the predicted results are usually use confusion matrix visualization. Confusion matrix uses value of accuracy for visualization. As shown in Figure 3, samples using neural network prediction: FP represents the number of other categories that are predicted to be the current category; TP represents the number of categories that are predicted to be correct; FN represents the number of current categories that are predicted to be other categories; FP represents the probability that other categories are predicted to be correct; and the final value will fill in the complete visual confusion matrix.

### 2.4 t-Distributed Stochastic Neighbor Embedding methods

T-sne was proposed by Laurens van der Maaten and Geoffrey Hinton in 2008. It is a machine learning algorithm mainly used for dimensionality reduction of data. After dimensionality reduction, data can be better visualized, so that experimental results can be observed and model parameters can be adjusted according to the experimental results. To improve model performance, T-sne was used to reduce the dimensionality of the data of the whole connection layer in the prediction process, and the relations between categories were represented by two-dimensional images (Li et al., 2020).

### 2.5 Process of exercise fatigue diagnosis

**FIGURE 4:**
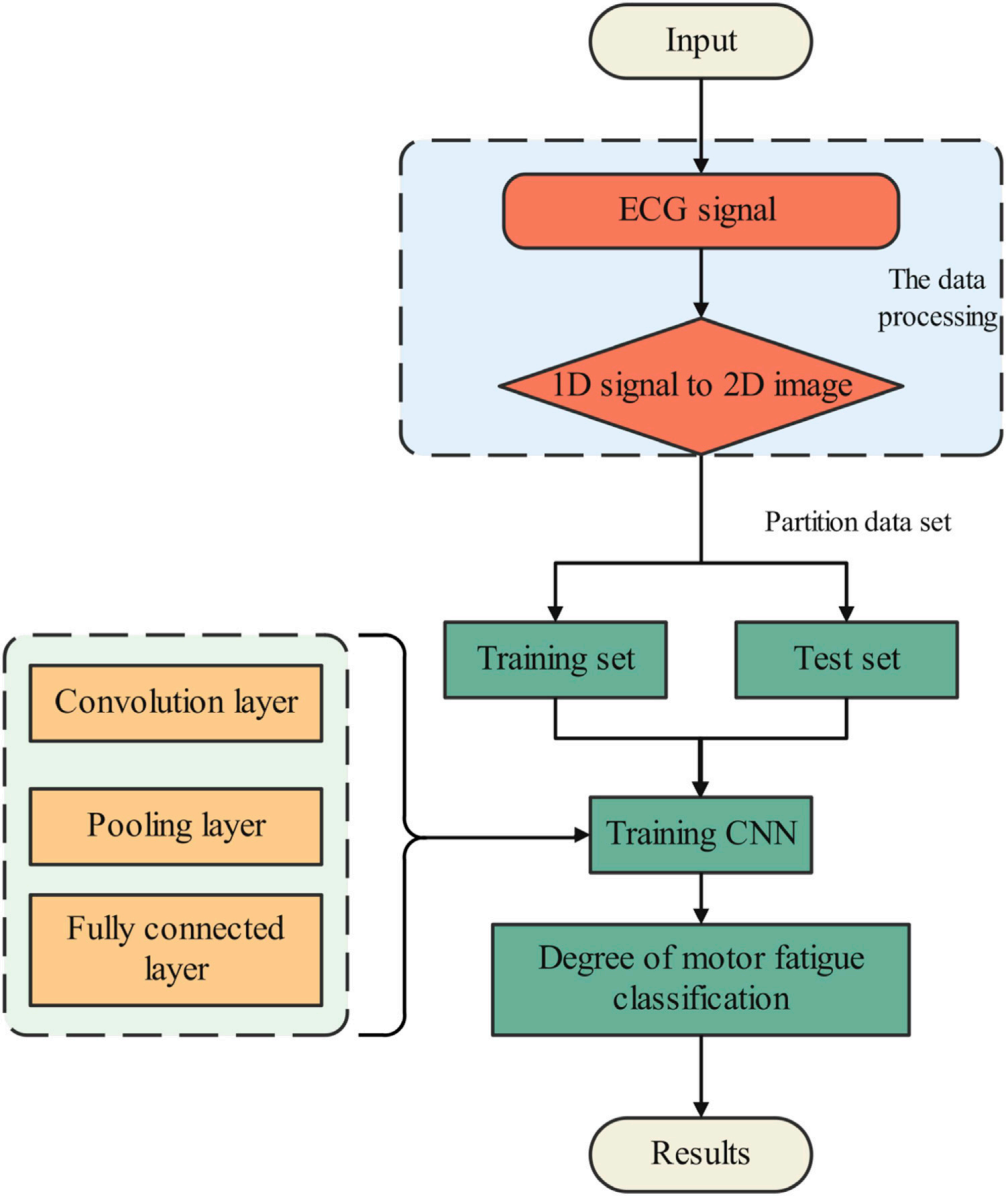
Overall flow chart.

The main process is to use VGG convolutional neural network for feature extraction and classification of ECG signals, and then detect the fatigue degree of human movement according to the trained model, as shown in Figure 4.

Step 1 First, ECG signals in motion are collected by ECG sensors, and then STFT is used to process the ECG raw data to obtain a two-dimensional spectrogram.

Step 2 The spectrum graph data set is divided into training set and verification set, and then the training set is fed to the VGG neural network for training.

Step 3 According to the obtained loss function, accuracy value and visualization results, the parameters of the VGG neural network model are constantly adjusted to make the neural network model convergent. Finally, the validation set is sent into the model for testing, and after satisfactory test results are obtained, the neural network model can be used for fatigue monitoring.

Step 4 The neural network model is applied to real movement fatigue monitoring.

## 3 Datasets

In this study, synchronous ECG and echocardiogram databases from Physical Web were used as validation data to verify the validity of the method (Goldberger, 2000; Kazemnejad et al., 2021). The dataset address is at www.physionet.org/content/ephnogram/1.0.0/. The biomedical engineering review committee of Shiraz University approved the acquisition experiment of ECG and PCG data. Before the experiment, each subject signed a written consent to participate in the study in learning about the subjects’ physical condition through structured interviews. According to the structured interview, the subjects can be determined with good physical condition, without cardiovascular diseases. Three hours before the test, the subjects’ diets are controlled, that is, they can drink, but eating and, alcohol intake and caffeinated beverages are banned. Then data are acquired. 24 male subjects between the ages of 23 and 29 took part in the experiment, and the experiment was conducted indoors.

**FIGURE 5:**
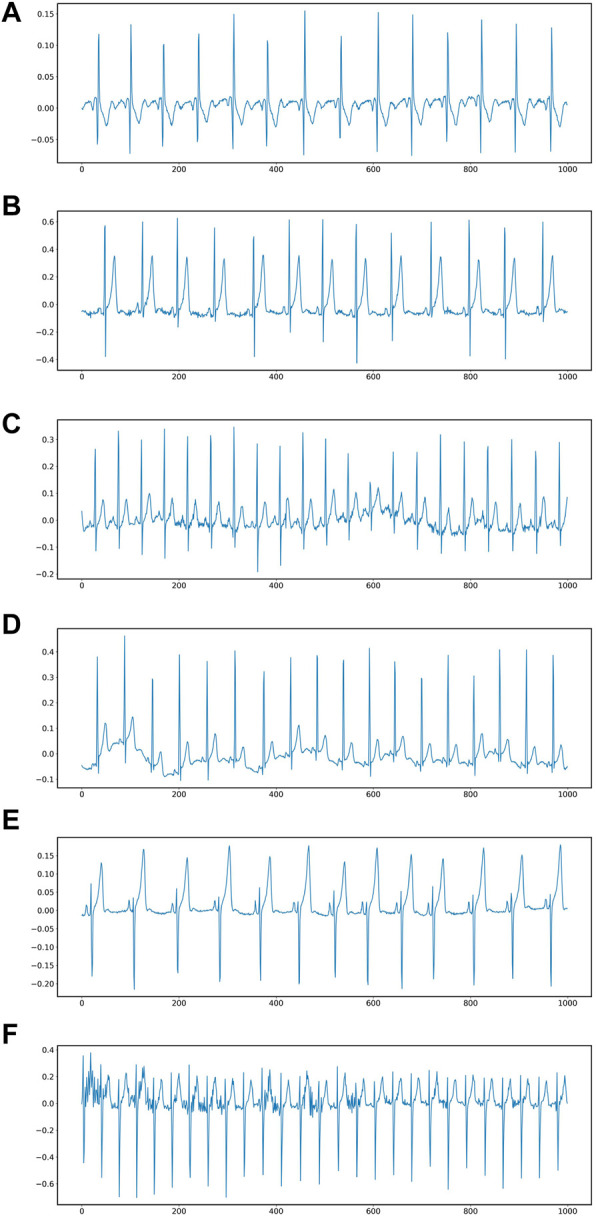
ECG at six different fatigue states: **(A)** lying in bed, **(B)** sitting in an armchair, **(C)** walking at a constant speed, **(D)** pedaling a stationary bicycle, **(E)** riding a stationary bicycle, **(F)** Bruce protocol treadmill stress test.

A 3-lead ECG acquisition device with a sampling rate of 8 kHz and a resolution of 12 bits was used. Each volunteer performed a specific task and 69 ECG and PCG recordings were collected. In the few cases where the data quality was poor, the test was repeated to obtain acceptable data. Poor quality samples were also included in the dataset for noise research purposes and marked as low quality in the spreadsheet accompanying the dataset. In our validation experiments, poor quality data were discarded for data balance. The data set was divided into six different fatigue levels according to the exercise in the test. 1) Fatigue level 1: ECG signal in a calm lying state. 2) Fatigue level 2: Subjects remained calm, sat in a chair, and did nothing else. 3) Fatigue level 3: Subjects performed light activity and walked at a constant speed of 3.7 km/h 4) Fatigue level 4: Subjects rode at a constant speed on a fixed bicycle. 5) Fatigue level 5: The subjects rode on a fixed bicycle, but increased the load until they were tired. 6) Fatigue level 6: Subjects were tested running at an increasing pace until they became fatigued. Figure 5 shows 6 different signal waveforms.

**FIGURE 6:**
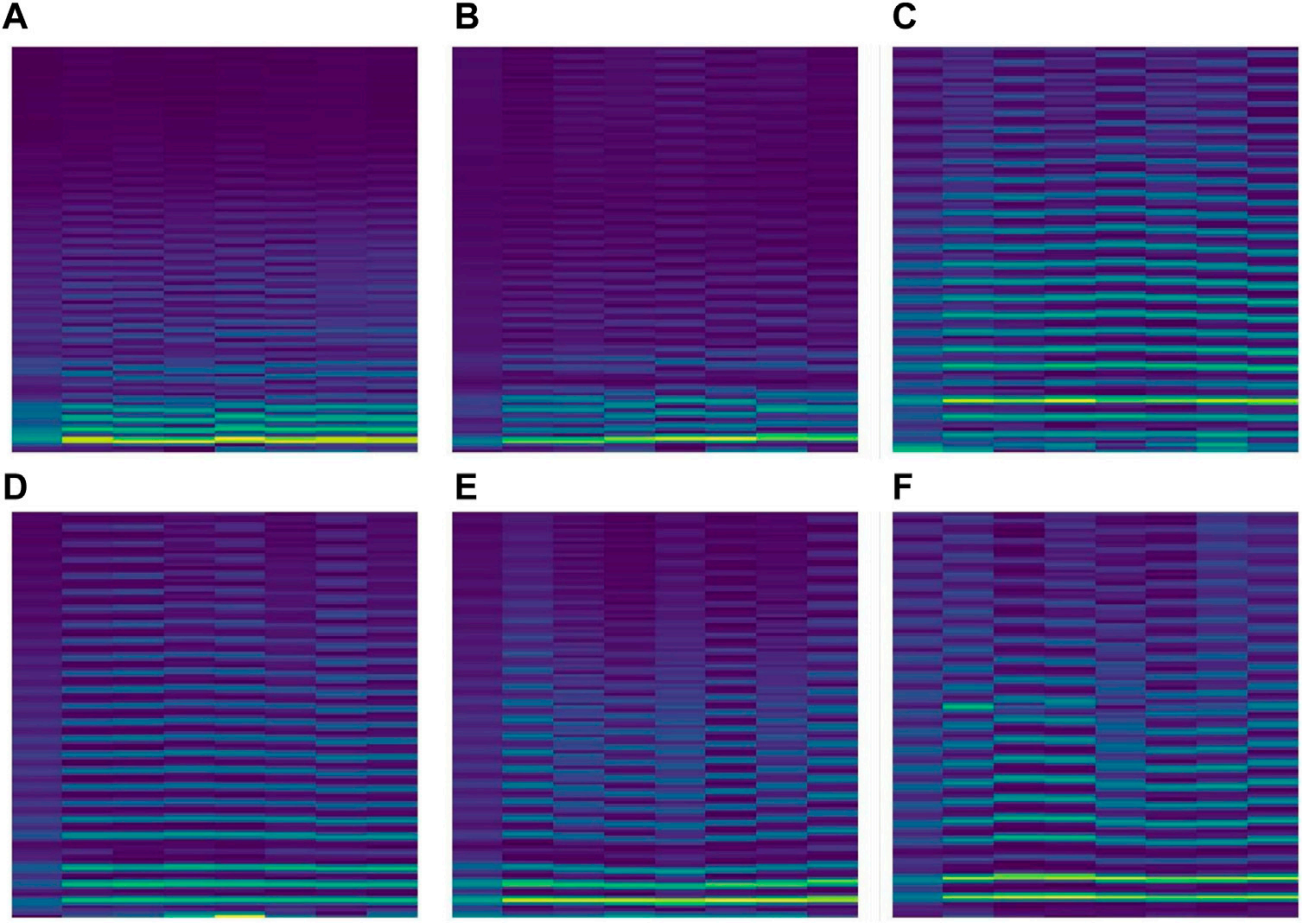
ECG spectrograms after STFT processing: **(A)** lying in bed, **(B)** sitting in an armchair, **(C)** walking at a constant speed, **(D)** pedaling a stationary bicycle, **(E)** bicycle stress test, **(F)** Bruce protocol treadmill stress test.

During the training, the data set was divided into 6 levels according to the motion state of different fatigue levels. The lowest level of fatigue was when the subject lay flat on the bed in a calm state, and the subject accelerated continuously until the tired ECG signal dataset was defined as the highest level of fatigue, at which the ECG signal would be identified as overtired and should be stopped for rest. Only records with a time of about 30 min are selected in the experiment. At the sampling frequency of 8KHZ, each data file contains about 14400,000 data points, which are too large for the experiment. In order to improve the calculation efficiency, the original data is down-sampled and the sampling frequency of the down-sampled data becomes 0.08khz. At this sampling frequency, the Numerical Python library function in Python is used to divide 1000 data points into a sample. Figure 6 is a data segment after segmentation. It can be seen that when 1000 data points are used as a sample, there are several cycles in a period of time, and the resulting spectrogram will better represent the fatigue state.

## 4 Experiments and analysis

The hardware device for the experiments was a Lenovo laptop with an Intel Core i7-10875H CPU and an Nvidia GeForce RTX 2060 GPU with 16 GB of RAM. The notebook uses a 64-bit Windows 10 operating system, and the system uses the python runtime environment and the keras deep learning framework to build the required CNN structure.

The spectrogram dataset of ECG signals will be divided into 90% training set and 10% test set. The number of training samples per iteration of the VGG model is 32, the number of training iterations is 40, and the learning rate is set to 0.0001.

### 4.1 Comparative experiment

The ECG data was processed using the STFT two-dimensional method, and the data set was divided according to the ratio of 1:9, and the sample data set shown in Table 1 was obtained. In order to compare the effectiveness of this method, three different two-dimension (2D) methods such as direct rendering method, GAF method and MTF method are also used. Direct drawing means that the ECG signal does not perform any processing, and directly uses the *plt* function in the matplotlib package in Python to convert it into a two-dimensional image. When it contains multi-channel signals, the ECG signal is fused into a two-dimensional image. The Gram Angular Difference Field (GAF) method encodes the time-domain signal by the Gram Angular Difference Field to generate a Gram Angular Field Image (GAF) containing motion fatigue features. The Markov transition field (MTF) coding method uses the MTF matrix to encode the time series into a two-dimensional image, which uniquely corresponds to the one-dimensional (1D) time series and contains the features in the time series (Xiao et al., 2020).

**TABLE 1:** The ECG signal is processed through an STFT data set.

Class	Trainset	Validation set
Fatigue level 1	387	42
Fatigue level 2	258	28
Fatigue level 3	387	42
Fatigue level 4	387	42
Fatigue level 5	387	42
Fatigue level 6	258	28

**FIGURE 7:**
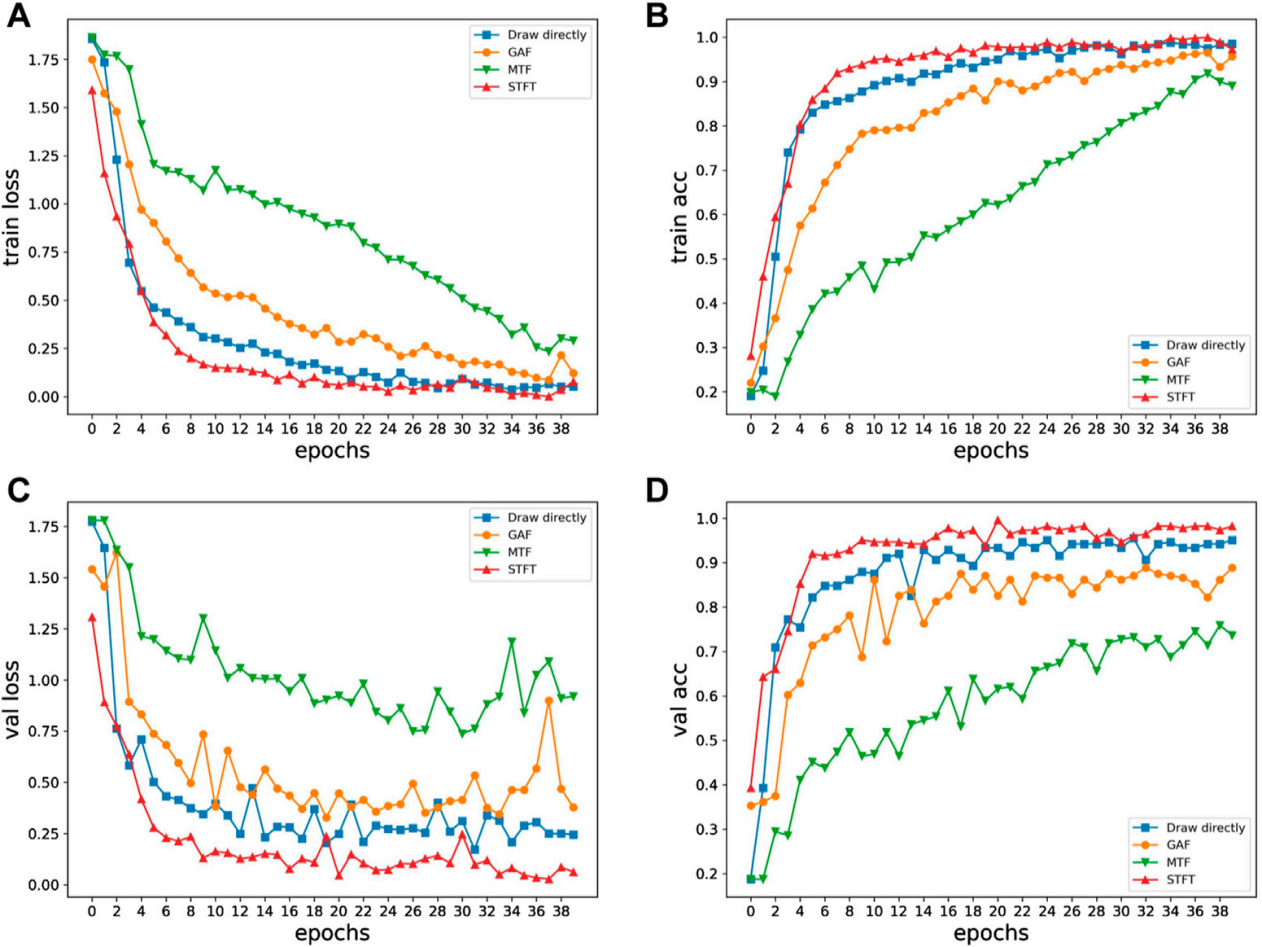
Loss and acc curves of four methods:**(A)** Train loss, **(B)** Train acc, **(C)** Val loss, **(D)** Val acc.

Four different 2D methods are trained using the same VGG neural network, and the training results for four different methods are presented in Figure 7. Figure 7 plots the final loss and accuracy after training on datasets generated by the four 2D methods. The direct drawing method got 93.3% accuracy on the validation set; the GAF method got 86.1% accuracy on the validation set; the MTF method got 73.6% accuracy on the validation set; the STFT method got 97.7% accuracy. It can be seen that the method of STFT processing ECG data achieves the lowest loss value and the highest accuracy on both the training set and the validation set. While identifying accurately, it has a high convergence speed.

### 4.2 Confusion matrix and cluster analysis

**FIGURE 8:**
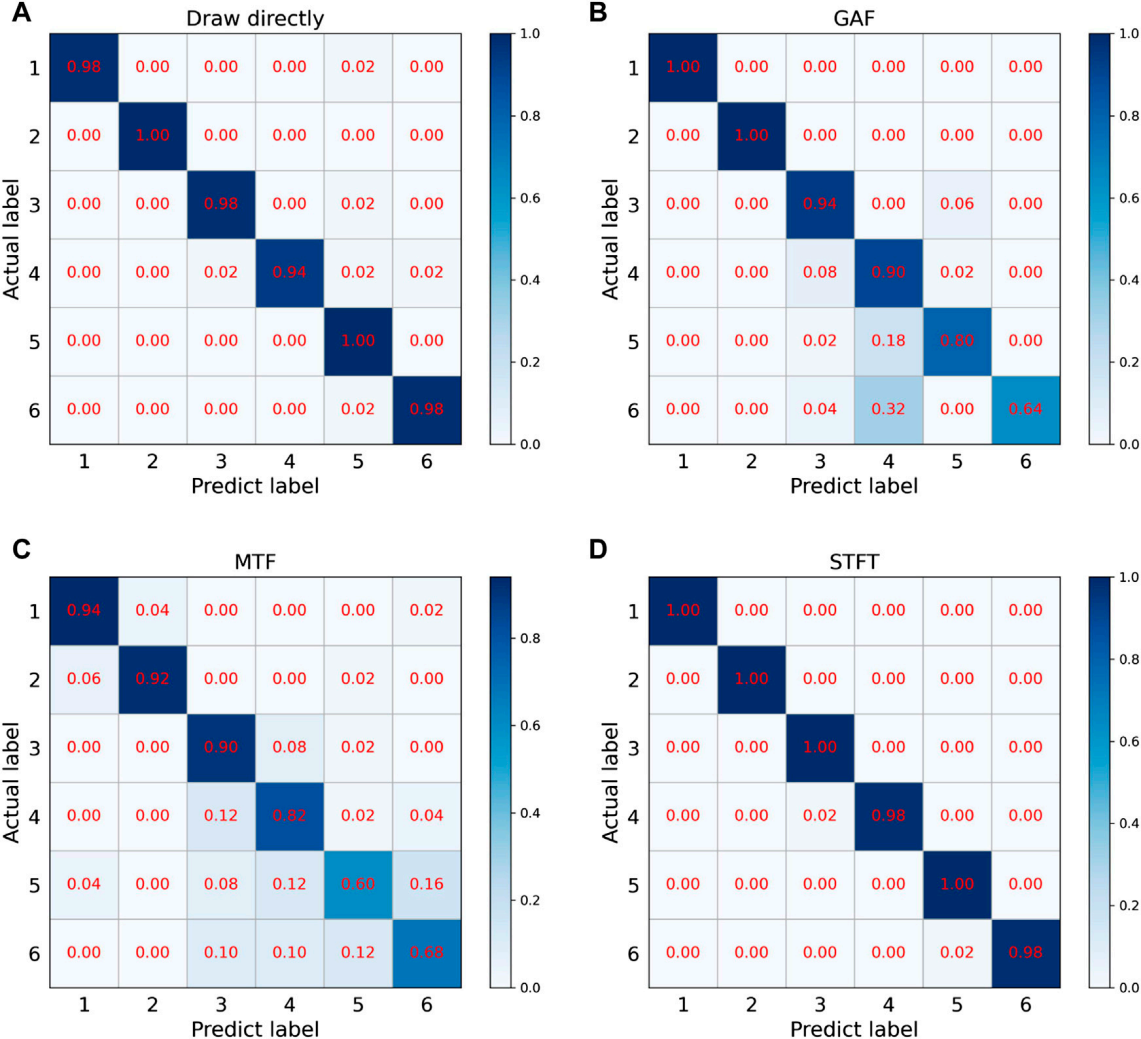
Confusion matrix of four methods:**(A)** Draw directly, **(B)** GAF, **(C)** MTF, **(D)** STFT.

**FIGURE 9:**
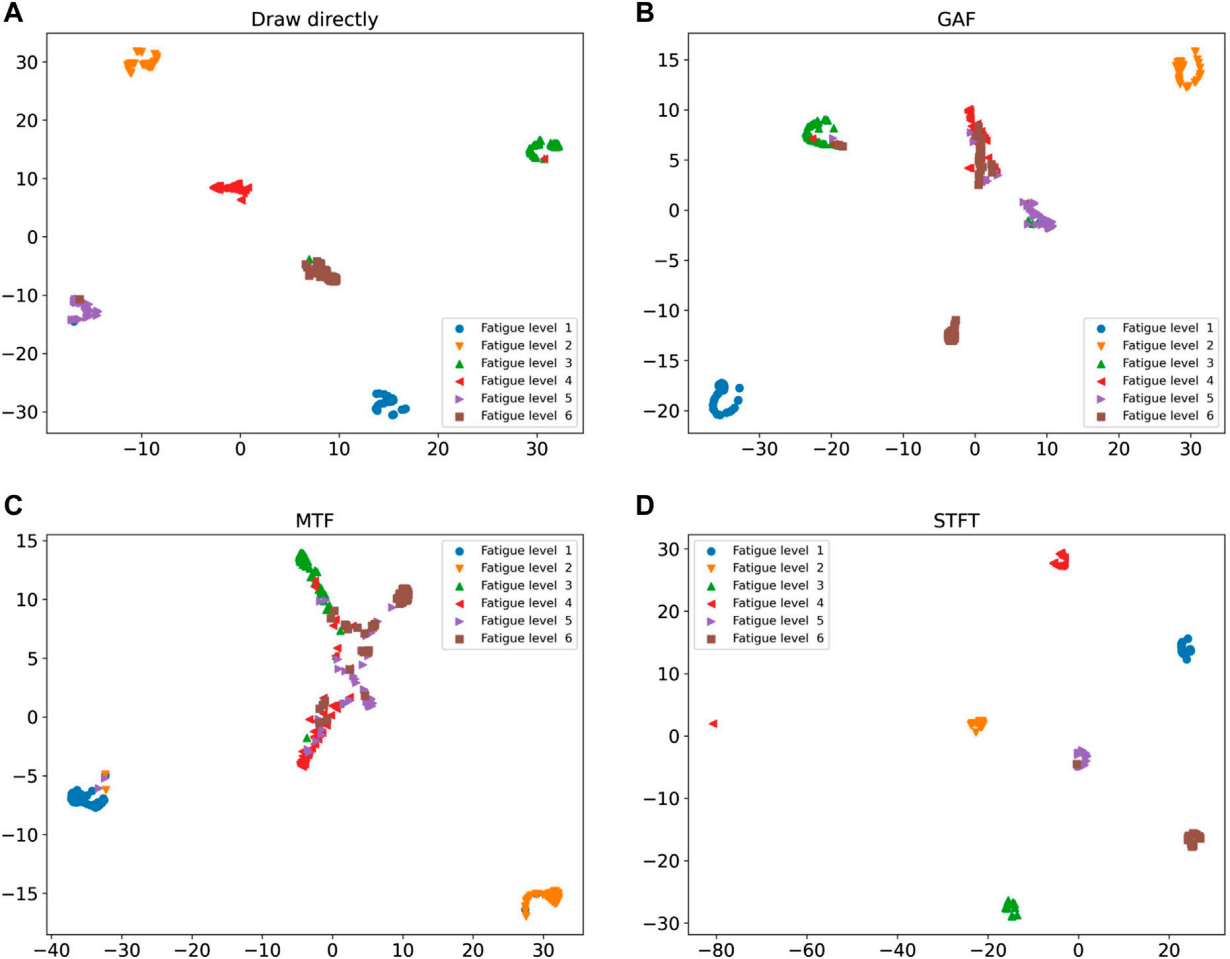
Cluster analysis of four methods:**(A)** Draw directly, **(B)** GAF, **(C)** MTF, **(D)** STFT.

The existing results cannot intuitively see the impact of different signal processing methods on fatigue recognition, so it is necessary to visualize the recognition results of the model on the validation set to better observe the classification results. The prediction results are shown in Figure 8 with the confusion matrix, and the fully connected layer data is shown in Figure 9 after dimensionality reduction using t-SNE. The GAF and MTF methods have larger errors in distinguishing higher fatigue levels in the confusion matrix. The proposed STFT method has the smallest error, only 2% of the 4th class is recognized as the 3rd class, and 2% of the 6th class is recognized as the 5th class, which may be due to a similar motion. Therefore, there is a similar ECG signal and degree of exercise fatigue, which leads to errors in the results.

The clustering results also prove that STFT and VGG neural networks have stronger classification performance. In Figure 9, the results obtained by GAF and MTF methods show that the distance between categories is close, and the categories are loose, while a large part of the categories are mixed together, so the effect is not ideal. In the direct rendering method, there are still many classification errors between categories. In STFT and VGG neural network methods, the clustering boundary is obvious; the classification errors are low; the distance between the categories is large; the inner classes are tight. Therefore, THE classification result of ECG signal processed by STFT in neural network is better than that of other data processing methods.

## 5 Conclusion

ECG signal is the physiological signal that can best reflect the fatigue state during exercise. ECG signals also have different characteristic axes under different exercise fatigue degrees and different fatigue states, so ECG signals have the characteristics of diversity and complexity. With the application of high-precision heart rate sensors in wearable devices, while traditional methods only use part of ECG signal information, it is difficult to meet the requirements of accurate monitoring in the face of these huge data. To solve these problems, a motion fatigue diagnosis method based on short-time Fourier transform and convolutional neural network was proposed, and the fast classification and diagnosis of ECG signals was realized by using VGG convolutional neural network. First, using STFT to process the 1D ECG signal to obtain a 2D spectrogram, then divide the data set, and use the VGG neural network for training and diagnosis. In this study, different two-dimensional processing methods were used for experiments. The combination of STFT and VGG neural network has the highest classification accuracy, and obtained a fatigue level diagnosis accuracy of 97.7%. Compared with the common 1D data processing method, it has the highest accuracy and the fastest convergence speed. It can be seen that when converting 1D time series into 2D images for fatigue level diagnosis, the STFT conversion method can effectively represent the characteristic information in the signal. The VGG network structure has better classification performance for fatigue diagnosis based on ECG signal. The combination of STFT data processing and VGG convolutional neural network can make full use of human ECG signals in exercise, reduce the complex process of extracting features, and quickly diagnose the current human fatigue level with strong robustness and effectiveness.

## Data Availability

Publicly available datasets were analyzed in this study. This data can be found here: https://www.physionet.org/content/ephnogram/1.0.0/.
